# Circulating Memory B Cells in Early Multiple Sclerosis Exhibit Increased IgA^+^ Cells, Globally Decreased BAFF-R Expression and an EBV-Related IgM^+^ Cell Signature

**DOI:** 10.3389/fimmu.2022.812317

**Published:** 2022-02-16

**Authors:** Jonatan Leffler, Stephanie Trend, Natalie C. Ward, Georges E. Grau, Simon Hawke, Scott N. Byrne, Allan G. Kermode, Martyn A. French, Prue H. Hart

**Affiliations:** ^1^ Telethon Kids Institute, University of Western Australia, Perth, WA, Australia; ^2^ Centre for Neuromuscular and Neurological Disorders, Perron Institute for Neurological and Translational Science, University of Western Australia, Perth, WA, Australia; ^3^ Dobney Hypertension Centre, Medical School, University of Western Australia, Perth, WA, Australia; ^4^ School of Medical Sciences, Faculty of Medicine and Health, The University of Sydney, Sydney, NSW, Australia; ^5^ Centre for Immunology and Allergy Research, Westmead Institute for Medical Research, Westmead, NSW, Australia; ^6^ Institute for Immunology and Infectious Disease, Murdoch University, Perth, WA, Australia; ^7^ School of Biomedical Sciences, University of Western Australia, Perth, WA, Australia; ^8^ Immunology Division, PathWest Laboratory Medicine, Perth, WA, Australia

**Keywords:** multiple sclerosis, B cells, memory B cells, IgA, BAFF, short chain fatty acids, Epstein Barr virus, flow cytometry

## Abstract

Multiple sclerosis (MS) is an immune-mediated inflammatory disease of the central nervous system that results in demyelination of axons, inefficient signal transmission and reduced muscular mobility. Recent findings suggest that B cells play a significant role in disease development and pathology. To further explore this, B cell profiles in peripheral blood from 28 treatment-naive patients with early MS were assessed using flow cytometry and compared to 17 healthy controls. Conventional and algorithm-based analysis revealed a significant increase in MS patients of IgA^+^ memory B cells (MBC) including CD27^+^, CD27^-^ and Tbet^+^ subsets. Screening circulating B cells for markers associated with B cell function revealed a significantly decreased expression of the B cell activation factor receptor (BAFF-R) in MS patients compared to controls. In healthy controls, BAFF-R expression was inversely associated with abundance of differentiated MBC but this was not observed in MS. Instead in MS patients, decreased BAFF-R expression correlated with increased production of proinflammatory TNF following B cell stimulation. Finally, we demonstrated that reactivation of Epstein Barr Virus (EBV) in MS patients was associated with several phenotypic changes amongst MBCs, particularly increased expression of HLA-DR molecules and markers of a T-bet^+^ differentiation pathway in IgM^+^ MBCs. Together, these data suggest that the B cell compartment is dysregulated in MS regarding aberrant MBC homeostasis, driven by reduced BAFF-R expression and EBV reactivation. This study adds further insights into the contribution of B cells to the pathological mechanisms of MS, as well as the complex role of BAFF/BAFF-R signalling in MS.

## Introduction

Multiple sclerosis (MS) is a chronic autoimmune disease of the central nervous system (CNS) characterized by inflammation, demyelination and neuronal damage (1). The disease usually starts with relapsing and remitting phases (RRMS) generally thought to be driven by inflammatory events maintained by activated macrophages, T cells and B cells that migrate into the CNS (2). Clinically isolated syndrome (CIS) is one of the earliest forms of MS and therefore, studies of circulating immune cells from CIS patients are important to understand pathogenesis of early disease and the development of CNS-autoimmunity.

B cells have emerged as a principal therapeutic target in people with MS as use of anti-CD20 monoclonal antibodies that deplete B cells, such as rituximab and ocrelizumab, have proven to be a very effective therapy (3). However, a major drawback of anti-CD20 therapy is that multiple B cell subsets are depleted and their long-term use may have adverse effects on the generation and maintenance of antibodies and memory B cells (MBCs) after infections or vaccinations, as exemplified by responses to SARS-CoV-2 vaccines (4). As existing antibody-producing plasma cells do not express CD20, these cells are likely not impacted by anti-CD20 therapy. Autoantibodies have been reported in MS patients, and extrafollicular immunoglobulin (Ig) deposits in the CNS are common and may contribute to disease progression (5). However, they are unlikely to be responsible for the rapid improvement in pathology following anti-CD20 therapy. This suggests that antibody-independent functions of B cells are likely also important in MS, including antigen presentation to CD4^+^ T cells and/or induction of “auto proliferative” Th1 CD4^+^ T cells (6), possibly facilitated by the HLA-DR15 gene variant (7). While uncertainty remains about which MBC subpopulations and functions might mediate these effects (8), one population that has emerged as being of particular interest is MBCs expressing the T-box family transcription factor (T-bet), the activity of which results in Th1-skewed immune responses and enhances control of infections by viruses and other intracellular pathogens (9, 10). Early reports highlighted that some MS patients exhibit an expansion of “age-associated B cells” including double negative (IgD^-^/CD27^-^) B cells as well as CD21^low^ B cells (11), which are now recognised to be primarily T-bet^+^ MBCs (10). More recently, T-bet^+^/CXCR3^+^ IgG1^+^ MBCs were found in the CSF, meninges and brain of patients with advanced MS, although their abundance in the circulation was decreased compared to controls (12). In contrast, an increased abundance of circulating T-bet^+^/CXCR3^+^ MBCs was observed in a small number of patients with early MS (13). In MS, induction of CXCR3^+^ MBCs appears to be associated with reactivation of Epstein Barr Virus (EBV) infection (14). As studies in mice have suggested that T-bet^+^ B cells are potent antigen-presenting cells for T cells and capable of producing Th1 cytokines, particularly IFN-γ and IL-12 (10, 15), it has been proposed that T-bet^+^ MBCs are preferentially recruited to the CNS of patients with MS and act as antigen presenting cells for pathogenic T cells (2).

B cells may also provide anti-inflammatory signals in the CNS as demonstrated by gut-derived IgA^+^ B cells that migrate into the CNS, first observed in the experimental autoimmune encephalomyelitis (EAE) model (16) and confirmed in the CNS of MS patients with active disease (17). IgA^+^ B cells are reactive with antigens of gut microbes, produce IL-10 and appear to display an antibody secreting cell phenotype (17). Further studies are needed to elucidate their contribution to, or importance for, controlling MS pathology. Their close correlation with gut homeostasis is notable as emerging evidence, including from our laboratory (18) suggests that gut homeostasis, microbial composition and circulating levels of short-chain fatty acids (SCFAs) are likely altered in MS patients and may also directly contribute to MS pathology (19, 20). However, whether or not this effect is mediated through IgA^+^ B cells remains to be determined, as is the potential therapeutic implications of these findings.

B cell maturation and differentiation is dependent on external survival signals such as the B cell activating factor (BAFF)/BAFF receptor (BAFF-R) pathway. Together with A proliferation-inducing ligand (APRIL), BAFF interacts with two related receptors; transmembrane activator and CAML interactor (TACI) and B-cell maturation antigen (BCMA), which are expressed at different stages of B cell differentiation (21). BAFF-R is essential for B cell survival, and is highly expressed on naive B cells, whereas the highest levels of TACI are found on MBCs. In addition to B cell depletion therapy, attempts to reduce B cell survival and modulate B cell function have been trialed in MS by inhibiting the activity of BAFF/APRIL using Atacicept, a soluble TACI receptor that binds both BAFF and APRIL and appears effective in reducing pathology in other autoimmune diseases such as systemic lupus erythematosus (22). However, in MS, the trial had to be aborted as patients treated with Atacicept experienced increased relapse frequency compared to placebo (23), suggesting that some BAFF/APRIL dependent B cell subsets may have beneficial effects on MS pathology.

In this study of both CIS and MS patients, the phenotypes of disease-associated circulating B cell subsets were investigated leading to the identification of increased circulating IgA^+^ MBCs in CIS/MS patients. Further analysis indicated these included distinct populations of T-bet^+^/CD21^low^, CD27^-^ and CD27^+^/CD24^hi^ IgA^+^ MBC populations. We also demonstrated that expression of BAFF-R was reduced on most B cell subsets of CIS/MS patients and observed phenotypical and functional associations with reduced B cell BAFF-R in CIS/MS. Finally, we determined if these abnormalities were associated with serological markers of EBV reactivation. While no associations were demonstrated with the abundance of IgA^+^ MBCs or BAFF-R expression, we did find that reactivation of EBV infection was associated with greater expression of HLA-DR and markers of a T-bet differentiation pathway amongst IgM^+^ MBCs, suggesting that EBV infection might influence the initiation of disease-associated B cell abnormalities in CIS/MS patients.

## Material and Methods

### Study Participants

Patients were recruited through neurology clinics in Perth and Sydney, Australia. Samples were also utilised from the PhoCIS trial as previously described (24). All were within 120 days of a well-defined, uni- or multi-focal demyelinating event. Patients were treatment naïve with no previous DMT treatment. Two patients were included who had had a short three- to five-day course of intravenous corticosteroids for their recent symptoms, this was followed by a one-month wash-out period before donation of the blood sample analysed in this study. Age and sex-matched controls were recruited in a separate study and only peripheral blood mononuclear cells (PBMC) were isolated. Demographics of the patients and controls included in the study are displayed in **Table 1**. The study was approved by the Sir Charles Gairdner Hospital Human Research Ethics committee (2006–073), Bellberry Human Research Ethics Committee (2014-02-083), the Human Research Ethics Office of the University of Western Australia (RA/4/1/6796) as well as from the University of Sydney (2018/377 and 2018/708). All participants provided their written informed consent to participate. This research was carried out in accordance with the recommendations of the National Health and Medical Research Council of Australia’s National Statement on Ethical Conduct in Human Research.

**Table 1:** Study Participant Characteristics.

Characteristics	CIS	MS	CIS/MS (combined)	Controls	Significance of difference CIS/MS compared to Controls (p)
n	15	13	28	17	
Females, n (%)	9 (60%)	11 (84.6%)	20 (71.4%)	9 (52.9%)	0.21 (Pearson)
Age, median (95% CI)	39.2 (23.4-54.3)	33.7 (20.3-52.1)	37.0 (20.3-54.3)	34 (21-54)	0.8 (Wilcoxon)
EBV status					
EBNA IgG Ab^+^, n (%)	15 (100%)	13 (100%)	28 (100%)	not available	not available
VCA IgM Ab^+^, n (%)	3 (20%)	4 (33.3%)	7 (25.9%)	not available	not available

### Sample Processing

Peripheral venous blood was collected into sodium heparin and SST vacutainers (BD Biosciences) to isolate PBMC and serum, respectively (25). For this study, PBMC were thawed from liquid N_2_ storage (25) and stained using custom-made antibody panels as described below.

### Serological Assays

Serum BAFF was measured using an enzyme-linked immunoassay according to the manufacturer’s instructions (R&D Systems) (25). Serum levels of antibodies against EBV nuclear antigen (EBNA)-1 (IgG) and EBV viral capsid antigen (VCA; IgM and IgG) were assessed by PathWest diagnostic laboratory (Perth, Western Australia) on serum from the CIS and MS patients (26).

### Short Chain Fatty Acid Analysis

Serum SCFAs were analyzed as previously described (27). Briefly, serum samples were spiked with ^13^C-sodium acetate, ^13^C-sodium butyrate and ^13^C-sodium propionate (all from Sigma) as internal standards, acidified and homogenized in isopropanol. Following centrifugation, the supernatant was collected and injected into an Agilent HP 6890 Series GC System, equipped with an Agilent 5973 Network Mass Selective Detector in splitless mode. Samples were separated on a DB Waxeter column (30 m x 0.25 mm x 0.25 μm), using a helium carrier gas at a flow rate of 1.0 ml/min. Identities and retention times of the SCFAs were established using the volatile-free acid mix and were manually integrated using the MSD ChemStation (version D.03.00.611). Concentrations were determined using the internal standard references and calculated as nanomoles per microlitre of serum.

### Cell Cultures

Data from short term cultures were utilized from previously conducted assays (25). Briefly, 800,000 cells/well were cultured in RPMI supplemented with 10% heat inactivated fetal calf serum (FCS), 5 μg/ml gentamicin, 2 mM L-glutamine, 50 μM 2β-mercaptoethanol and 1 μg/ml GolgiPlug (BD Biosciences) in the presence of 1 μg/ml R848 (In vivoGen) for 18 h at 37°C and 5% CO_2_. Following culture, samples were transferred into flow cytometry tubes and stained as per below using an antibody panel as defined in **Supplementary Table 1**.

### Flow Cytometry

For *ex vivo* staining, freshly thawed PBMC, 500,000/sample, were washed in PBS, stained for viability with FVS780 (BD Biosciences) for 10 min, blocked with 4% FCS in PBS for 5 min, separated by centrifugation at 500 g for 5 min and resuspended in extracellular antibody cocktails (**Supplementary Tables 2, 3**) at 4°C for 30 min. Following incubation, samples were washed with 1 ml of 4% FCS in PBS, centrifuged and resuspended in 4% FCS in PBS with the relevant streptavidin conformation if required (**Supplementary Table 3**), for 20 min at 4°C. Following incubation, samples were washed and centrifuged as above followed by resuspension in Foxp3 Transcription Factor Staining Buffer (eBioscience) or Cytokine fix (BD Bioscience, **Supplementary Table 3**) for 30 min at 4°C, followed by another wash step. Samples were then resuspended in the relevant intracellular antibody cocktail and incubated for 30 min at 4°C followed by a wash. Samples were finally resuspended in 4% FCS in PBS, stored for <24 h at 4°C and acquired using a LSR Fortessa (BD Biosciences). For analysis, samples were compensated in FlowJo 10.5.3 (BD Biosciences) and gated as depicted in **Supplementary Figure 1**. Expression levels were assessed on a minimum of 10 events. For analysis in R, compensated files from the Live/Lin^-^/CD19/20^+^ or Live/Lin^-^/CD19/20^+^/IgA^+^/IgD^-^ populations were exported from FlowJo into R 4.1.1 (R Core Team) and R Studio 1.4.1717 (Rstudio PBC) using *FlowCore* 2.5.0 (28). FlowSOM clustering, UMAP visualization and cluster annotations were performed using the *CATALYST* 1.17.3 package (29).

### Statistical Analysis

The statistical significance of difference between two groups was calculated using Mann-Whitney’s test; for comparison of multiple experimental groups, Kruskal-Wallis test was used followed by Dunn’s uncorrected post-test. Significance of correlations between two continuous parameters was assessed using Spearman’s non-parametric test. For comparison of categorical data between groups, Pearson’s χ^2^ test was used; all statistical analysis was performed using JMP 15.2 (SAS Institute Inc.) and visualized using Prism 8.02 (GraphPad Software).

## Results

### Increased Abundance of Circulating IgA^+^ MBC in CIS/MS Patients

**Figure 1:**
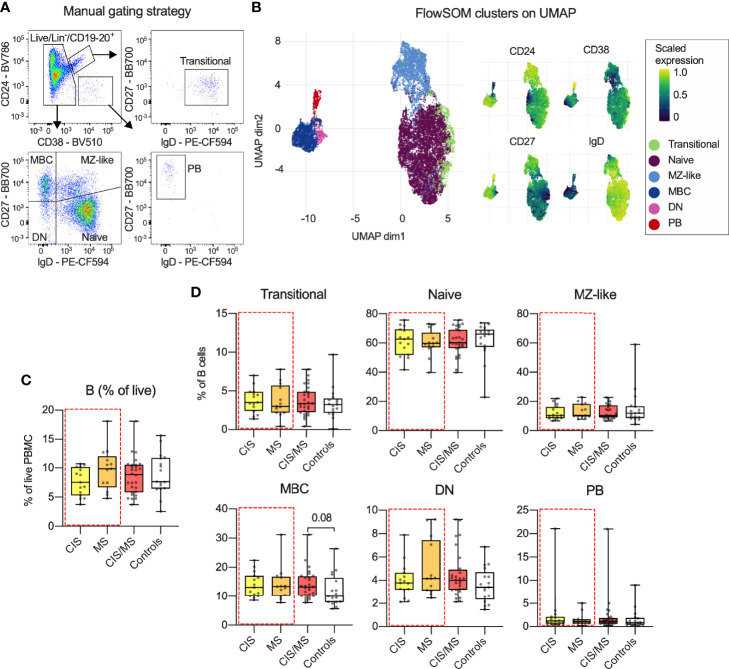
**(A)** Gating strategy and B cell subset definitions. **(B)** Clusters identified using FlowSOM clustering and UMAP-based visualization on the total B cell population as well as expression of relevant markers across each cluster. **(C, D)** Abundance of total B cells **(C)** and proportion of B cell subsets **(D)** within the B cell population in CIS, MS, combined CIS/MS and compared to controls. Data in **(A)** are representative from one individual and in **(B)** a representative subset of 33 study participants. Data in **(C, D)** are displayed for each participant, together with box/whiskers display for median and range (n _CIS_ = 15, n _MS_ = 13, n _Controls_ = 17). Significance of difference between patient groups versus controls was calculated using Mann-Whitney non-parametric test and displayed as respective p value.

To identify CIS- or MS-specific B cell profiles in blood, three custom-made flow cytometry panels were used (**Supplementary Tables 2, 3**). These contained antibodies to a set of core markers (**Supplementary Table 2**) to identify transitional, naive, marginal zone-like (MZ-like), memory B cells (MBC), double negative (DN) and plasmablast (PB) B cell subsets. These subsets were identified using both manual gating (**Figure 1A** and **Supplementary Figure 1**) and confirmed using unsupervised FlowSOM clustering and visualized using UMAP (**Figure 1B**). All analyses were carried out using data derived from manual gating. By comparing the median abundance of total B cells (**Figure 1C**) as well as the six subsets (**Figure 1D**) in data from the three panels, no differences were observed in CIS/MS patients (25), nor in the CIS or MS groups analyzed separately (demographic data in **Table 1**), compared to healthy controls. However, there was a trend towards increased abundance of MBC in CIS/MS patients compared to controls (p = 0.08).

**Figure 2:**
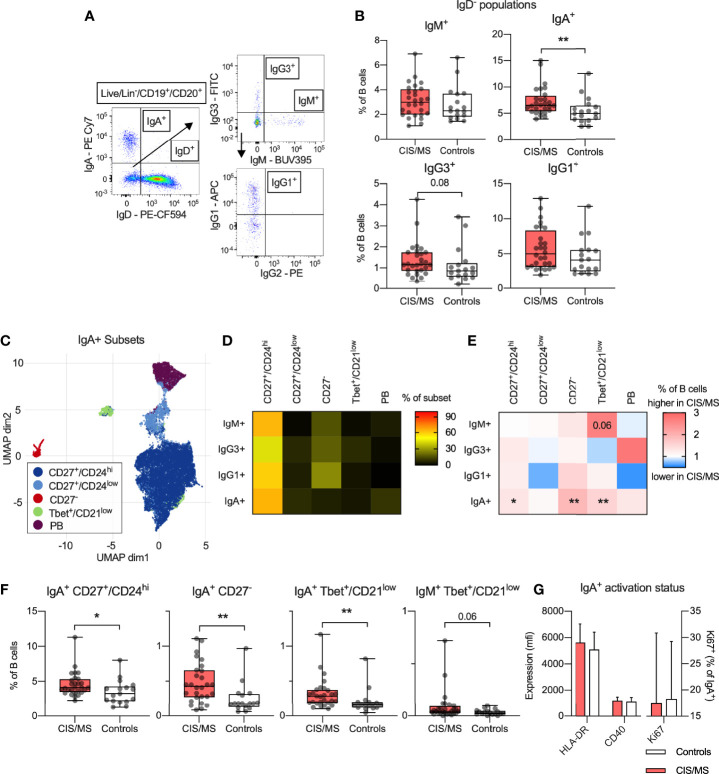
**(A)** Gating strategy of isotype specific B cell subsets. **(B)** Abundance of IgM^+^, IgA^+^, IgG3^+^ and IgG1^+^ MBC within the B cell population in CIS/MS patients compared to controls. **(C)** Clusters identified using FlowSOM clustering and UMAP-based visualization on IgA^+^ MBC. **(D)** Abundance of identified subset within each isotype specific MBC. **(E, F)** Differences in mean ratio in CIS/MS compared to controls **(E)** for each subset as well as raw data for significantly different subsets **(F)**. **(G)** Expression of HLA-DR or CD40 on IgA^+^ MBC as well as proportion of Ki67^+^ IgA^+^ MBC in CIS/MS and controls. Data in A are representative from one individual, data in **(D, E, G)** are displayed as means of CIS/MS/Controls combined **(D)**, ratios of means **(E)** or mean ± SD **(G)**. Data in F are displayed for each participant, together with box/whiskers display for median and range (n _CIS/MS_ = 28, n _Controls_ = 17). Significance of difference between patient groups versus controls was calculated using Mann-Whitney non-parametric test on the raw data and displayed as p<0.01, **, p<0.05, * or for p<0.1, as specific p-value.

To further characterise the MBC populations, memory B cell subsets, as defined by their loss of IgD expression (IgD^-^) were next assessed. B cells were classified based on expression of immunoglobulin (Ig) isotypes IgM, IgG3, IgG1 and IgA prior to gating by CD27 expression as shown in **Figure 1A**. Although antibodies to IgG2 were included in the panel, unsatisfactory staining was obtained and IgG2^+^ subsets were left out of the analysis (**Figure 2A**). The abundance of IgA^+^ B cells in CIS/MS patients was significantly increased compared to controls, and there was also a trend towards an increased proportion of IgG3^+^ B cells circulating in CIS/MS patients compared to controls (**Figure 2B**) no difference between CIS and MS patients was observed (**Supplementary Figure 2**). To further investigate if there were any phenotypic differences in the IgA^+^ B cell subset, as well as in other subsets, FlowSOM clustering was used to identify Ig isotype-specific subsets expressing B cell differentiation markers. The IgA^+^ subset separated into five main clusters (**Figure 2C**) and based on their expression of differentiation markers (**Supplementary Figure 3A**) they were annotated as CD27^+^/CD24^hi^, CD27^+^/CD24^low^, CD27^-^, Tbet^+^/CD21^low^ and CD27^+^/CD38^hi^ (PB) B cells. Varied CD24 expression has previously been observed across memory populations although the biological impact remains unclear (30). These B cell subsets were also identified using manual gating (**Supplementary Figure 3B**). Applying this gating to each Ig-specific subset, we observed that, as expected, all subsets were dominated by the CD27^+^/CD24^hi^ population (**Figure 2D**). By comparing the abundance of each Ig-specific subset between CIS/MS patients and controls, the CD27^-^ and Tbet^+^/CD21^low^ IgA^+^ were increased two-fold in CIS/MS patients, and a minor increase in the CD27^+^/CD24^hi^ subset was also observed compared to controls. No difference was observed for the minor CD27^+^/CD24^low^ and PB subsets (**Figures 2E, F**), suggesting that expansion of IgA^+^ MBC is not uniform. There was also a trend towards an increased proportion of Tbet^+^/CD21^low^ cells within the IgM^+^ MBC subset in CIS/MS patients compared to controls (**Figures 2E, F**), no difference between CIS and MS patients was observed for any of these subsets (**Supplementary Figure 4**). Upon assessment of markers associated with activation on IgA^+^ MBC, no differences were observed in expression of HLA-DR, CD40 or Ki67^+^ in CIS/MS compared to controls (**Figure 2G**). Together these data suggest that the increase in IgA^+^ MBC abundance is reflected across multiple IgA^+^ subsets and that there are no differences in IgA^+^ MBC activation status.

### IgA^+^ MBC and Serum Levels of Short Chain Fatty Acids

**Figure 3:**
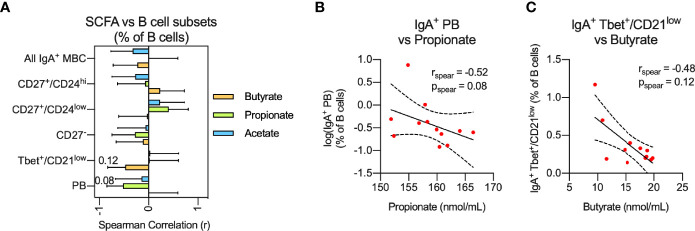
**(A, B)** Non-parametric correlation between plasma levels of the SCFAs; Butyrate, Propionate and Acetate with abundance of IgA^+^ MBC or IgA^+^ MBC subsets **(A)** as well as display of raw data for the two strongest correlations **(B)** identified in **(A)**. Correlations are visualized using a linear fit and 95% CI. Data are displayed as mean correlation ± 95% CI **(A)** or for each participant (n = 12) in **(B)**. Significance of correlation was calculated using Spearman non-parametric test displayed as specific p-value.

Circulating IgA^+^ MBC were significantly increased in CIS/MS patients compared to controls. Given that IgA^+^ MBC were recently reported to be increased in the CNS of MS patients and displayed specificity against commensal micro-organisms in the intestines (17), an association was investigated between the abundance or phenotype of circulating IgA^+^ B cells, and serum levels of SCFAs (18). Although no statistically significant associations were observed, a trend for two negative correlations was noted between serum levels of propionate and the proportion of IgA^+^ PBs (p=0.08; **Figures 3A, B**) as well as butyrate and the proportion of IgA^+^ Tbet^+^/CD21^low^ MBCs (p=0.12; **Figures 3A, C**) in CIS/MS patients. This result suggests that reduced levels of SCFA may be related to increased levels of circulating IgA^+^ subsets in CIS/MS patients (**Figures 2E, F**).

### B Cell Expression of BAFF-R Is Decreased in CIS/MS Patients

**Figure 4:**
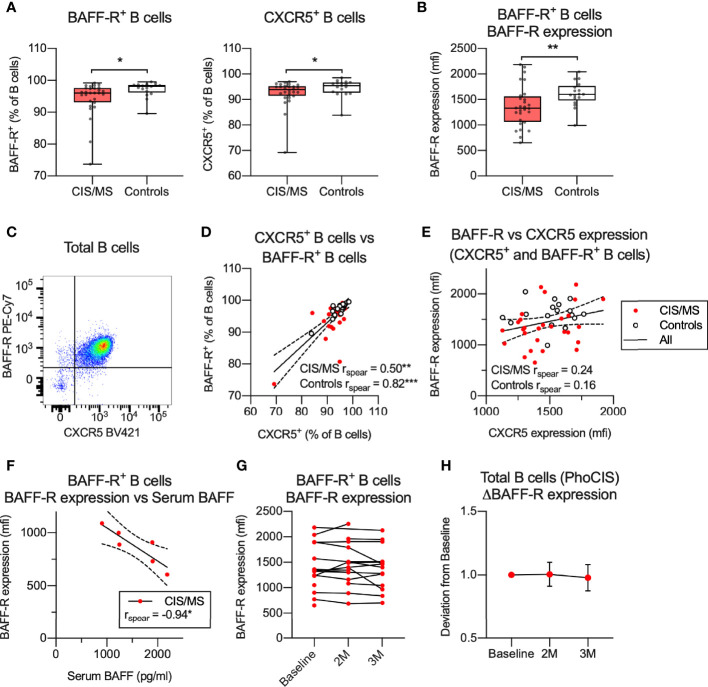
**(A, B)** Abundance **(A)** or expression levels **(B)** of BAFF-R and/or CXCR5 on total or BAFF-R^+^ B cells. **(C)** Expression of BAFF-R and CXCR5 on total B cells. **(D)** Correlation of BAFF-R^+^ and CXCR5^+^ B cells in CIS/MS patients and controls. **(E)** Correlation of expression levels of BAFF-R and CXCR5 on BAFF-R^+^ and CXCR5^+^ B cells in CIS/MS patients and controls. **(F)** Correlation of BAFF-R expression on BAFF-R^+^ B cells and serum BAFF levels. **(G, H)** BAFF-R expression on BAFF-R^+^ B cells at study recruitment and at 2 months (2M) and 3 months (3M) follow-up, displayed as raw data **(G)** or mean ± SD change from baseline **(H)**. Data in **(A, B**, **D, E)** are displayed for each participant, together with box/whiskers display for median and range where relevant (n _CIS/MS_ = 28, n _Controls_ = 17). Data in **(C)** is displayed from a representative individual and in **(F)** from a subset of individuals (n _CIS/MS_ = 6). In **(G, H)**, data is displayed for (n _Baseline_ = 15, n _2M_ = 15, n _3M_ = 14). Correlations are visualized using a linear fit and 95% CI. Significance of difference between patient groups versus controls was calculated using Mann-Whitney non-parametric test on the raw data, correlation was calculated using Spearman non-parametric test and displayed as p<0.001, ***; p<0.01, **; p<0.05, *.

To further investigate potential B cell dysfunction in CIS/MS patients, selected markers of B cell function and/or activation were examined in patients and controls across B cell subsets, gated as per **Figure 1** (**Supplementary Figures 5A**–**C**). The results were expressed as the proportion of positive subsets or the level of marker expression, depending on whether the marker was expressed on a discrete population or as a continuum (**Supplementary Figure 5A**), and examined for associations with CIS/MS (**Supplementary Figures 5D, E**). Through this screen, differential expression of BAFF-R, CXCR5 and CD69 was identified across several B cell subsets. Due to low number of events, the difference in expression of CD69 on CD69^+^ PB was not investigated further. Positivity for BAFF-R and CXCR5 was decreased in the total B cell population from CIS/MS patients (**Figure 4A**) and expression levels (mfi) of BAFF-R were also decreased across B cells that were BAFF-R^+^ (**Figure 4B**). No difference was observed between CIS and MS patients, although a trend towards reduced CXCR5^+^ was observed in MS compared to CIS patients (**Supplementary Figures 6A, B**). In the combined groups, the majority of B cell subsets were positive for both BAFF-R and CXCR5 (**Figure 4C**) and most cells that were BAFF-R^+^ were also CXCR5^+^ in both CIS/MS patients and controls (**Figure 4D**). However, levels of BAFF-R and CXCR5 expression (mfi) did not appear associated (**Figure 4E**), suggesting that although the markers were expressed on the same B cell subset, levels of expression are likely regulated independently. We therefore focused the subsequent analyses on the level of BAFF-R expression rather than abundance of BAFF-R^+^ cells.

BAFF-R is activated through binding to its ligand BAFF, which leads to BAFF-R shedding and thus downregulation of surface BAFF-R expression (31). Increased serum BAFF levels and decreased BAFF-R expression on B cells was recently observed in interferon-β or fingolimod treated MS patients (32). Serum levels of BAFF were therefore assessed in a subset of the CIS/MS patients investigated in this study, who were all treatment-naïve. A strong negative correlation was observed between serum BAFF and levels of BAFF-R expression (**Figure 4F**). To determine if the decreased BAFF-R expression is stable over time, BAFF-R levels were assessed in samples collected longitudinally from CIS patients participating in the PhoCIS trial (24) and a limited change in BAFF-R expression was observed over a three-month study period (**Figures 4G, H**). Together these data suggest that the decreased BAFF-R expression observed in B cells from CIS/MS patients is related to serum levels of BAFF and that this phenotype is stable over time.

### Relationships Between BAFF-R Expression on B Cells and the Abundance of Circulating B Cell Subsets Are Abnormal in CIS/MS Patients

**Figure 5:**
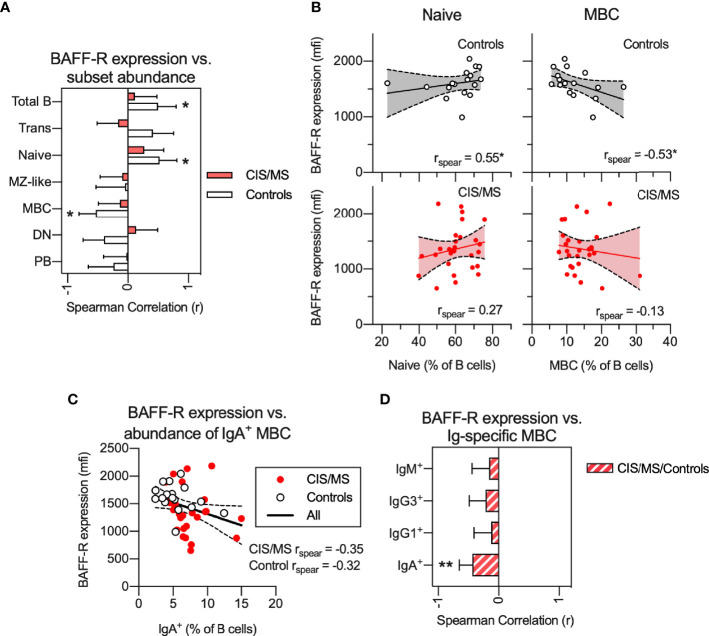
**(A, B)** Non-parametric correlation between BAFF-R expression on BAFF-R^+^ B cells and abundance of B cell subsets in CIS/MS patients and controls **(A)** as well as display of raw data for correlations with abundance of Naive and MBCs in CIS/MS and controls **(B)**. **(C)** Correlation of BAFF-R expression on BAFF-R^+^ B cells and abundance of IgA^+^ MBC. **(D)** Non-parametric correlation between BAFF-R expression on BAFF-R^+^ B cells and abundance of isotype specific MBC in the combined group of CIS/MS patients and controls. Data are displayed as mean correlation ± 95% CI **(A, D)** or for each participant (n _CIS/MS_= 28, n _Controls_ = 17) in **(B, C)**. Correlations are visualized using a linear fit and 95% CI. Significance of correlation was calculated using Spearman non-parametric test displayed as p<0.01, **; p<0.05, *.

Signaling from BAFF-R is essential for B cell survival and differentiation. In healthy controls, we observed a significant positive correlation of BAFF-R expression with abundance of total B cells (**Figure 5A**) and naive B cells (**Figures 5A, B**) as well as a negative correlation with the proportion of MBC (**Figures 5A, B**). In contrast, in patients with CIS/MS, no correlation between BAFF-R expression and proportions of any B cell subset was observed (**Figures 5A, B**). For comparison, there was a trend towards increased expression of TACI, another B cell receptor that binds BAFF, in CIS/MS patients compared to controls (**Supplementary Figure 7A**). However, expression of TACI did not correlate with abundance of any B cell subsets, although a weak negative correlation was observed with abundance of total B cells in CIS/MS patients (**Supplementary Figures 7B, C**). To assess if decreased BAFF-R expression was related to the increased abundance of IgA^+^ MBC observed in **Figure 2**, BAFF-R expression was correlated with abundance of IgA^+^ MBC (**Figure 5C**), without observing any significant correlation in either CIS/MS or controls. As correlation coefficients were comparable, the CIS/MS and control groups were combined to gain statistical power, and a significant correlation of BAFF-R expression with the abundance of IgA^+^ MBC was observed (**Figure 5D**). No correlation was observed for other Ig-specific MBC (**Figure 5D**) suggesting that BAFF-R expression may be specifically related to IgA^+^ MBC abundance.

### Decreased BAFF-R Expression on B Cells of CIS/MS Patients Is Associated With Higher TNF Production in Activated B Cells

**Figure 6:**
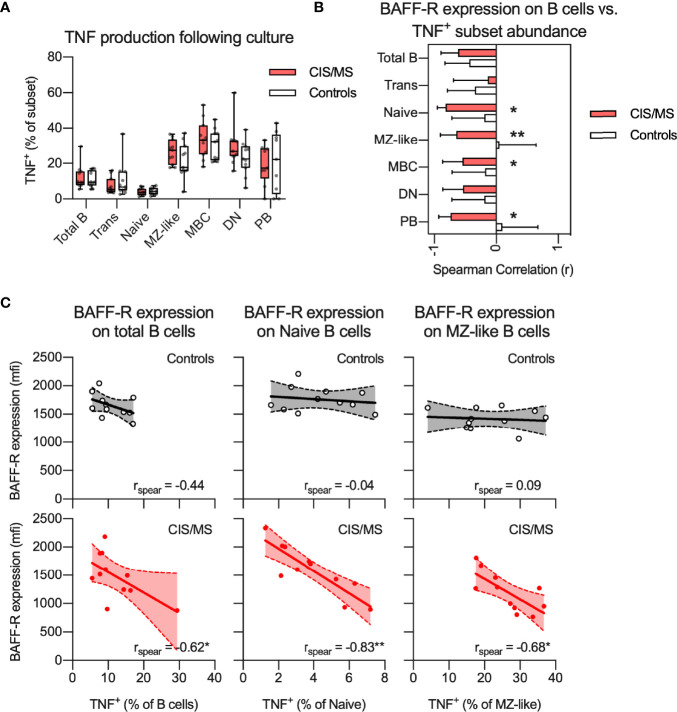
**(A)** Proportion of TNF^+^ B cell subsets following stimulation with R848 in CIS/MS patients and controls. **(B, C)** Non-parametric correlation between BAFF-R expression on BAFF-R^+^ B cells and proportion of TNF^+^ B cell subsets following R848 stimulation in CIS/MS patients and controls **(B)** as well as raw data for CIS/MS patients and controls **(C)**. Data in A and C are displayed for each participant, together with box/whiskers display for median and range where relevant (n _CIS/MS_ = 11, n _Controls_ = 11). Data in **(B)** are displayed as mean correlation ± 95% CI. Correlations are visualised using a linear fit and 95% CI. Significance of correlation was calculated using Spearman non-parametric test displayed as p<0.01, **; p<0.05, *.

To assess if reduced BAFF-R expression on B cells of CIS/MS patients was associated with abnormalities of B cell activation, BAFF-R expression on total B cells was correlated with TNF production, assessed in an 18 h culture system using the TLR7/8 agonist, R848, in the presence of a GolgiPlug agent (25, 33). There was no difference in R848-induced TNF^+^ cells from CIS/MS patients compared to those from controls across any B cell subset (**Figure 6A**). However, in patients with CIS/MS, there was a strong negative correlation between total B cell BAFF-R expression and TNF production (% of subset) in total B cells, naive B cells, MZ-like cells as well as in PB (**Figure 6B**), which was not observed for cells from the controls. These correlations were also interrogated using subset specific BAFF-R expression and the findings were confirmed (**Figure 6C**). As PB did not express BAFF-R, these were excluded from the analysis. This outcome suggested that in CIS/MS, decreased BAFF-R expression is associated with increased R848-induced TNF production. However, in controls, BAFF-R expression has no impact on TNF production.

### EBV Re-Activation in CIS/MS Patients Relates to a Specific IgM^+^ MBC Phenotype but Was Not Associated With BAFF-R Expression or Abundance of IgA^+^ MBC

**Figure 7:**
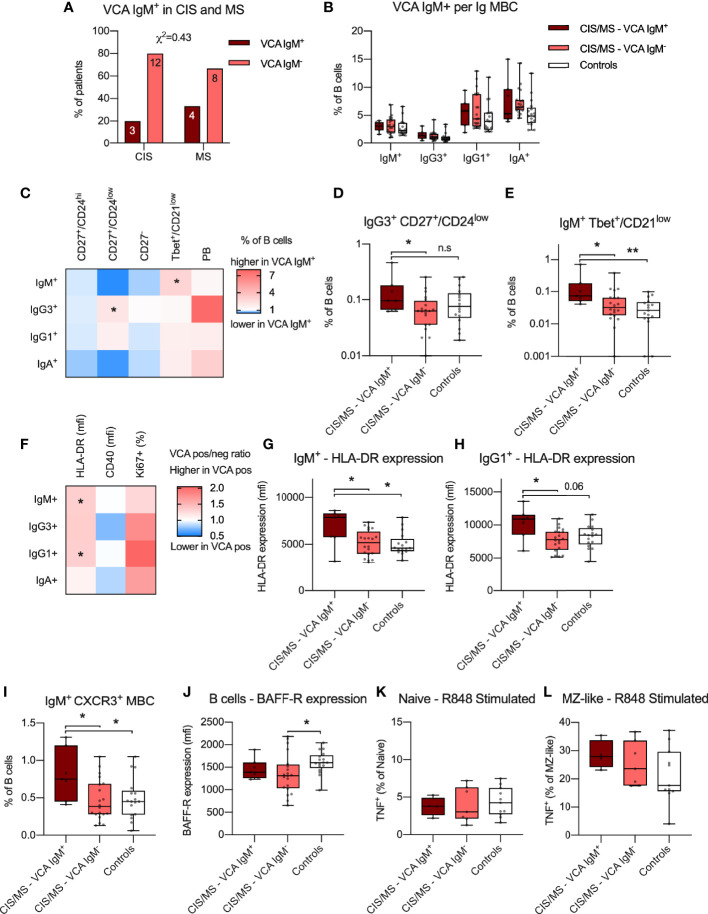
**(A)** Proportion of VCA IgM^+^ individuals with CIS or MS. Abundance of IgM^+^, IgG3^+^, IgG1^+^ and IgA^+^ MBC within the B cell population in VCA IgM^+^, VCA IgM^-^ CIS/MS patients as well as controls. **(C–E)** Differences in mean ratio in VCA IgM^+^ compared to VCA IgM^-^ CIS/MS patients **(C)** for each MBC subset as well as raw data for significantly different subsets **(D, E)**. **(F–H)** Differences in mean ratio in VCA IgM^+^ compared to VCA IgM^-^ CIS/MS patients for expression of HLA-DR, CD40 as well as proportion of Ki67^+^ MBC within each MBC subset **(F)** as well as raw data for significantly different subsets **(G, H)**. **(I)** Proportion of IgM^+^ CXCR3^+^ MBC in VCA IgM^+^, VCA IgM^-^ and controls. **(J)** Expression of BAFF-R on BAFF-R^+^ B cells in VCA IgM^+^ and VCA IgM^-^ CIS/MS and controls. **(K, L)** Proportion of TNF^+^ Naive **(K)** and MZ-like **(L)** B cells following R848 stimulation in VCA IgM^+^ and VCA IgM^-^ CIS/MS patients and controls. Data in **(A)** are displayed as proportion and number of CIS and MS patients. Data in **(B, D, E, G–L)** are displayed for each participant, together with box/whiskers display for median and range (n _VCA IgM Ab+_ = 7, n _VCA IgM Ab-_ = 20, n _Controls_ = 17) and in K-L (n _VCA IgM Ab+_ = 4, n _VCA IgM Ab-_ = 7, n _Controls_ = 11). Data in **(C, F)** are displayed as means of VCA IgM^+^ and VCA IgM^-^ ratios. Significance of difference between distribution of individuals was calculated using the χ^2^ test. For comparisons across multiple groups a Kruskal-Wallis test followed by a post test was used, for comparison between two groups Mann-Whitney’s non-parametric test was used. All statistical analysis was performed on the raw data and significance of differences is displayed as p<0.01, **, p<0.05, * or for p<0.1, as specific p-value; n.s., not significant.

Given that EBV infection has been associated with the pathogenesis of MS (34) and EBV establishes a persistent infection in B cells, reactivation of EBV may associate with phenotypical and functional abnormalities in B cells of CIS/MS patients (25). Markers of B cell differentiation and/or activation were therefore compared in CIS/MS patients with and without serum IgM antibodies (Ab) to EBV viral capsid (VCA) antigen, used as a marker of recent EBV re-activation (VCA IgM Ab^+^). As expected, all patients had previously had EBV infection as indicated by positivity for EBNA-specific IgG Ab (**Table 1**). In addition, 7 (26%) had evidence of a recent EBV re-activation as indicated by positivity for VCA IgM Ab (**Table 1**). Proportions of patients who were VCA IgM Ab^+^ were similar between the CIS and MS groups (**Figure 7A**) and samples were therefore analyzed together as previously. The abundance of MBC expressing different Ig isotypes was first compared between VCA IgM Ab^+^ and Ab^-^ patients without observing any difference (**Figure 7B**). However, there was an increased abundance of CD27^+^/CD24^low^ cells within the IgG3^+^ MBC subset in VCA IgM Ab^+^ patients when compared with VCA IgM Ab^-^ patients (**Figures 7C, D**), and an increased abundance of Tbet^+^/CD21^low^ cells within the IgM^+^ MBC subset in VCA IgM Ab^+^ patients when compared to VCA IgM Ab^-^ patients and controls (**Figures 7C, E**).

To evaluate if EBV reactivation was associated with differential expression of the functional markers HLA-DR and CD40 or proportion of Ki67^+^ cells, these markers were assessed across each Ig isotype-specific B cell subset (**Figure 7F**). In VCA IgM Ab^+^ patients, expression of HLA-DR was increased amongst IgM^+^ and IgG1^+^ MBCs when compared to both VCA IgM Ab^-^ patients and controls (**Figures 7F–H**), though the difference was not statistically significant compared to controls for IgG1^+^ MBC (**Figure 7H**). Based on the impact that EBV reactivation appeared to have on IgM^+^ MBC, we further assessed if the abundance of CXCR3^+^ IgM^+^ MBCs was increased as previously observed (14) and could confirm a significantly increased proportion of CXCR3^+^ IgM^+^ MBC in VCA IgM Ab^+^ individuals compared to VCA IgM Ab^-^ CIS/MS patients or controls (**Figure 7I**). The increase in IgM^+^ Tbet^+^/CD21^low^ cells and increased expression of HLA-DR appeared dependent on recent EBV re-activation as no significant association was observed with titres of EBNA IgG or VCA IgG levels (**Supplementary Figures 8A, B**). However abundance of IgG3^+^ CD27^+^/CD24^low^ and IgM^+^ CXCR3^+^ MBC correlated weakly with EBNA IgG and VCA IgG titres respectively (**Supplementary Figures 8C, D**), suggesting these markers may also relate to the initial response to EBV infection. To determine if EBV re-activation impacted BAFF-R expression, BAFF-R expression was compared between VCA IgM Ab^+^ and IgM Ab^-^ patients with no significant difference observed, although decreased BAFF-R expression in the VCA IgM Ab^-^ patients compared with controls was observed (**Figure 7J**). No difference was also observed in TNF production by naive or MZ-like cells following R848 stimulation in VCA IgM Ab^+/-^ patients (**Figures 7K, L**). No correlation with EBNA or VCA IgG titres was observed for BAFF-R expression or R848 induced TNF responses (**Supplementary Figures 8E**–**G**). Together, these data suggest that reactivation of EBV infection in CIS/MS patients is associated with an altered B cell phenotype, particularly in IgM^+^ MBC. However, markers of EBV reactivation do not appear to be associated with the CIS/MS-associated B cell phenotype of increased abundance of IgA^+^ MBC and reduced BAFF-R expression as identified in this study.

## Discussion

The role that B cells play in the immunopathogenesis of MS remains elusive. In the current study we have examined the circulating MBC compartment of early MS patients compared to controls. Using expression of Ig isotypes in the BCR (IgD^-^ and either IgM^+^, IgG3^+^, IgG1^+^ or IgA^+^) to define MBCs, we have demonstrated that CIS/MS patients exhibit three abnormalities that may be relevant to understanding how B cell dysfunction contributes to the immunopathogenesis of MS. Firstly, we demonstrated expansion of IgA^+^ MBCs, which included 3 distinct populations: a major CD27^+^/CD24^hi^ population and two smaller populations of CD27^-^ or T-bet^+^/CD21^low^ cells. There was also a trend towards expansion of T-bet^+^ IgM^+^ MBCs. Secondly, we demonstrated decreased expression levels of BAFF-R, which was apparent on all BAFF-R expressing subsets. Importantly, reduced BAFF-R expression was associated with altered B cell homeostasis and activation. Finally, we also observed that within the CIS/MS group, EBV VCA IgM positivity was associated with increased HLA-DR expression and expression of markers of a T-bet^+^ differentiation pathway, including CXCR3, on IgM^+^ MBCs.

Our demonstration that circulating IgA^+^ MBCs are expanded in patients with early MS is, to our knowledge, a novel finding. These cells might be the gut-microbiota-specific IgA^+^ B cells with putative regulatory activity as recently described (17) and/or a response to disturbances in the gut microbiome composition. Gut microbiome dysbiosis has been reported in MS patients, with microbiome metabolites, including SCFAs, able to regulate reactivity of immune cells and their function in the CNS. In particular, propionate, butyrate and acetate are recognized to be anti-inflammatory in function and in various disease settings, may induce regulatory immune cells (35). In the current study, weak associations (p<0.1) were observed between IgA^+^ PB and serum propionate levels as well as Tbet^+^/CD21^low^ IgA^+^ MBC and serum butyrate levels. Propionate was previously identified to be significantly reduced in the serum of our CIS/MS cohort (18). Others have also identified low propionate levels in MS patients and supplementation alleviated their disease symptoms (36), as reviewed (37). In our study, the functions of circulating IgA^+^ MBC were not analyzed and further studies are required. Gut-derived acetate and butyrate have been shown to promote B regulatory cells through independent mechanisms (38, 39). Importantly, no definitive marker of B regulatory cells has been described. Instead, many B regulatory cells are pleiotropic and their function (the production of IL-10, IL-35, TGFβ) is likely determined dose-dependently by their environment, and the need for immune homeostasis (40).

The second major observation from this study is the decreased expression of BAFF-R across multiple B cell subsets in CIS/MS patients compared to controls. BAFF-R is part of the BAFF-APRIL system which consists of the two signaling molecules BAFF and APRIL and their receptors; BAFF-R, TACI and BCMA. In the current study, BAFF-R expression was closely associated with serum BAFF levels, supporting previous observations that BAFF-R is downregulated in response to circulating levels of BAFF (41). Several studies have reported that serum levels of BAFF are increased in MS patients (42, 43) or are increased in the CNS (44), potentially due to increased production of BAFF by EBV-infected B cells (45), although elevated BAFF is not observed in all studies (46). In experimental EAE, BAFF contributes to EAE pathology, through both B and T cell dependent mechanisms (47) and a BAFF antagonist also effectively reduced manifestations of EAE (48). However, the BAFF/BAFF-R contribution to MS pathology remains unclear. Observations have suggested that IFN-β treatment, that reduces MS pathology, increases BAFF levels (49). In a longitudinal study, MS patients with the highest levels of BAFF appeared to be the least likely to experience an exacerbation (43) suggesting that increased BAFF levels in MS may not necessarily contribute to disease. Further supporting a protective role of BAFF in MS are findings from clinical trials of Atacicept, which was administered to extend the time between relapses but increased the relapse frequency in the intervention group, even though mature B cell numbers and serum Ig concentrations decreased significantly (23). The mechanisms for this increase in relapse remain unknown, but may be related to off-target effects on TACI signaling or APRIL. In patients with leukemia, increased BAFF leads to increased IL-10^+^ B cells through TACI (50). Further studies are currently underway using Belimumab, a monoclonal antibody specifically to BAFF to exclude reduction of APRIL signaling (NCT04767698).

In the current study, decreased BAFF-R expression on B cells of CIS/MS patients remained stable over at least three months and was associated with altered B cell differentiation and activation. In controls, BAFF-R expression was closely associated with the proportions of circulating naive B cells and MBCs; however, in CIS/MS patients, this association was absent suggesting that the effects of BAFF/BAFF-R in maintaining homeostatic B cell differentiation may be impaired; whether this promotes survival of autoreactive MBC remains to be explored. In addition to associations with altered B cell development, associations were also observed with functional outcomes, in this case assessed by TNF production following R848 exposure *in vitro*. Although TNF production did not differ between CIS/MS patients and controls, we recorded a strong negative correlation between BAFF-R expression and TNF production, particularly in naive and MZ-like B cells in CIS/MS patients. This correlation was completely absent in controls. Together these data suggest that in CIS/MS, BAFF-R expression shifts from being associated with naive and MBC B cell differentiation to instead be associated with how B cells respond to stimulation and pre-disposition to pro-inflammatory activation, that may contribute to MS pathology. We have previously observed an inverse association with serum BAFF and expression of the inhibitory B cell receptor CD32b (25) and we are currently investigating the mechanisms of this disease specific signaling pathway further. A weak correlation between BAFF-R expression and increased abundance of IgA^+^ MBC was observed when all samples were analyzed together, however the mechanisms or relevance of this link are not clear. In a recent study on SLE patients treated with anti-BAFF therapy, the authors did observe an increase in IgA^+^ MBC in a subset of individuals (51). Further, mice deficient in BAFF-R display reduced levels of all immunoglobulins except IgA (52) suggesting that IgA^+^ MBC and IgA^+^ PB are generated through a BAFF-R independent pathway.

There is growing evidence that many B cell abnormalities associated with the immunopathogenesis of MS are related to EBV infection of MBCs (34). While we did not demonstrate a relationship between EBV reactivation and expansion of circulating IgA^+^ MBCs or decreased expression of BAFF-R on B cells, we did demonstrate a relationship with increased expression of HLA-DR molecules and markers of a T-bet differentiation pathway, including CXCR3, on IgM^+^ MBCs. It has been proposed that T-bet^+^ IgG^+^ MBCs expressing CXCR3 are induced by reactivation of EBV infection and preferentially recruited to the CNS of patients with MS where they act as APCs for pathogenic T cells (2, 12, 14). Tbet^+^/CD21^low^ MBC are a distinct population of germinal centre-experienced B cells that differentiate under the influence of T cells and Th1-associated cytokines, such as IFN-γ, and by stimulation *via* TLR-7 and -9, both of which are sensors of viral nucleic acids (10, 53, 54). Increased production of circulating T-bet^+^ MBC is part of the B cell response to infection by viruses and to viral vaccines (55). Furthermore, T-bet expression by human MBCs facilitates Ig isotype switching to IgG3 and IgG1 (10). In addition to our finding that reactivation of EBV infection was associated with expansion of circulating T-bet^+^ IgM^+^ MBCs, we also demonstrated an association with other MS-related MBC phenotypes. Specifically, we observed an increased abundance of CD27^+^/CD24^low^ IgG3^+^ MBCs and increased expression of HLA-DR on IgM^+^ and IgG1^+^ MBC. We have previously shown that circulating IgG3^+^ MBCs are expanded in patients with MS, though most subsets were CD24^+^ (56), and that serum IgG3 levels predicted the time of progression from CIS to MS (26). Increased HLA-DR expression on MBCs may facilitate B cell-mediated T cell activation as demonstrated in MS (6). As the findings on CD27^+^/CD24^low^ IgM^+^ and IgM^+^ CXCR3^+^ MBC also associated with EBNA IgG and VCA IgG titres, it is likely that these subsets may also be influenced by the initial response to EBV infection. Nevertheless, our findings provide further evidence that differentiation of B cells along a T-bet^+^ pathway may be related to reactivation of EBV infection and that this is turn may help to activate pathogenic T cells in CNS lesions of patients with MS.

In summary, we have demonstrated a previously unrecognized complexity of the circulating memory B cell compartment of patients with early MS. This includes evidence of an increased abundance of IgA^+^ MBCs that may be modulated by SCFAs, persistent reduction in expression of BAFF-R associated with abnormalities of B cell homeostasis and activation, and an EBV-related disease signature characterized by expression of HLA-DR and markers of a T-bet^+^ differentiation pathway in IgM^+^ MBC. Our findings provide evidence that reactivation of EBV infection in MS patients might program the early differentiation of MBCs towards a pathway that supports T cell activation, likely in an episodic manner, and that this is mainly observed in IgM^+^ MBCs. In contrast, decreased BAFF-R expression affected multiple B cell subsets, was persistent over time and not related to reactivation of EBV, possibly indicating that it is an effect of persistently increased BAFF production by pathogenic B cells in the CNS, which are reportedly infected by EBV (45). Similarly, the increased abundance of circulating IgA^+^ MBCs was not related to reactivation of EBV infection and may provide further evidence of a link between the mucosal immune system of the gut and the immunopathogenesis of MS (57).

## Data Availability Statement

The raw data supporting the conclusions of this article will be made available by the authors, without undue reservation.

## Ethics Statement

The studies involving human participants were reviewed and approved by Sir Charles Gairdner Hospital Human Research Ethics Committee, Bellberry Human Research Ethics Committee, Human Research Ethics Office of the University of Western Australia and Human Research Ethics Office of University of Sydney. The patients/participants provided their written informed consent to participate in this study.

## Author Contributions

JL, ST, and NCW performed the experiments. JL, ST, MAF, and PHH interpreted the data. GEG, SH, SNB, and AGK recruited patients and collected clinical data. JL, MAF, and PHH wrote and revised the manuscript. PHH supervised the study. All authors contributed to the article and approved the submitted version.

## Funding

The study was supported by funding from MSWA and MS Australia as well as the National Health and Medical Research Council of Australia for funding the PhoCIS trial (ID 1067209).

## Conflict of Interest

The authors declare that the research was conducted in the absence of any commercial or financial relationships that could be construed as a potential conflict of interest.

## Publisher’s Note

All claims expressed in this article are solely those of the authors and do not necessarily represent those of their affiliated organizations, or those of the publisher, the editors and the reviewers. Any product that may be evaluated in this article, or claim that may be made by its manufacturer, is not guaranteed or endorsed by the publisher.
