# Supplementary material for: Circulating Memory B Cells in Early Multiple Sclerosis Exhibit Increased IgA+ Cells, Globally Decreased BAFF-R Expression and an EBV-Related IgM+ Cell Signature
Source: Front Immunol. 2022 Feb 16;13:812317. doi: 10.3389/fimmu.2022.812317 (PMC8888440; doi:10.3389/fimmu.2022.812317)
Supplement: Supplementary file 1 [file DataSheet_1.docx]

Supplementary Material

**Circulating Memory B Cells in Early Multiple Sclerosis Exhibit Increased IgA^+^ Cells, Globally Decreased BAFF-R Expression and an EBV-Related IgM^+^ Cell Signature**

Jonatan Leffler^1^, Stephanie Trend^1,2^, Natalie C Ward^3^, Georges E Grau^4^, Simon Hawke^4^, Scott N Byrne^4,5^, Allan G Kermode^2,6^, Martyn A French^7,8^, Prue H Hart^1^

^1^Telethon Kids Institute, University of Western Australia, Perth, WA, Australia

^2^Centre for Neuromuscular and Neurological Disorders, Perron Institute for Neurological and Translational Science, University of Western Australia, Perth, WA, Australia

^3^Dobney Hypertension Centre, Medical School, University of Western Australia, Perth, WA, Australia^.^

^4^The University of Sydney, School of Medical Sciences, Faculty of Medicine and Health, Sydney, NSW, Australia

^5^Westmead Institute for Medical Research, Centre for Immunology and Allergy Research, Westmead, NSW Australia

^6^Institute for Immunology and Infectious Disease, Murdoch University, Perth, WA, Australia

^7^School of Biomedical Sciences, University of Western Australia, Perth, WA, Australia

^8^Immunology Division, PathWest Laboratory Medicine, Perth, WA, Australia

**Supplementary Figures and Tables**

**Table 1**

**B cell culture panel**

| **Antigen** | **Fluorochrome/Channel** | **Vendor** | **#Cat** | **Step** |
| --- | --- | --- | --- | --- |
| FVS575 | BV605 | BD Biosciences | 565694 | Viability, PBS |
| CD19 | BUV737 | BD Biosciences | 612756 | Extra cellular  PBS + 4% FCS |
| CD20 | BUV737 | BD Biosciences | 564432 |  |
| CD38 | BV510 | BD Biosciences | 563251 |  |
| CD24 | BV785 | BD Biosciences | 311142 |  |
| CD27 | BB700 | BD Biosciences | 746084 |  |
| IgD | PE-CF594 | BD Biosciences | 562540 |  |
| IgM | BUV395 | BD Biosciences | 563903 |  |
| CD64 | BV711 | BD Biosciences | 740782 |  |
| CD32b | AF488 | BioInvent International AB | n/a |  |
| TNF | PE Cy7 | BD Biosciences | 560678 | Intra cellular  Cytokine fix |
| IL-10 | PE | BD Biosciences | 559330 |  |

**Table 2**

**B cell core panel**

| **Antigen** | **Fluorochrome/Channel** | **Vendor** | **#Cat** | **Step** |
| --- | --- | --- | --- | --- |
| FVS780 | APC-Cy7 | BD Biosciences | 565388 | Viability, PBS |
| CD19 | BUV737 | BD Biosciences | 612756 | Extra cellular  PBS + 4% FCS |
| CD20 | BUV737 | BD Biosciences | 564432 |  |
| CD38 | BV510 | BD Biosciences | 563251 |  |
| CD24 | BV785 | BioLegend | 311142 |  |
| CD27 | BB700 | BD Biosciences | 746084 |  |
| IgD | PE-CF594 | BD Biosciences | 562540 |  |
| IgM | BUV395 | BD Biosciences | 563903 |  |
| CD3 | AF700 | BD Biosciences | 557917 |  |
| CD56 | AF700 | BD Biosciences | 557919 |  |
| CD14 | AF700 | BD Biosciences | 557923 |  |

**Table 3**

**B cell panel 1-3**

| **Panel** | **Antigen** | **Fluorochrome/**  **Channel** | **Vendor** | **#Cat** | **Step** |
| --- | --- | --- | --- | --- | --- |
| 1 | IgG1 | Biotin & S-APC | Miltenyi Biotec | 130-119-858 | Extra cellular  PBS + 4% FCS |
| 1 | IgG2 | PE | SouthernBiotech | 9060-09 |  |
| 1 | IgG3 | FITC | SouthernBiotech | 9210-02 |  |
| 1 | IgA | PE-Vio770 | Miltenyi Biotec | 130-113-477 |  |
| 1 | CD21 | BV650 | BD Biosciences | 742762 |  |
| 1 | HLA-DR | PE-Cy5 | BD Biosciences | 555813 |  |
| 1 | CD40 | BV605 | BioLegend | 334336 |  |
| 1 | Tbet | BV421 | BD Biosciences | 563318 | Intra cellular  FoxP3 transcription fix |
| 1 | Ki67 | BV711 | BioLegend | 350516 |  |
| 2 | IgG1 | Biotin & S-APC | Miltenyi Biotec | 130-119-858 | Extra cellular  PBS + 4% FCS |
| 2 | IgG3 | FITC | SouthernBiotech | 9210-02 |  |
| 2 | CD21 | BV650 | BD Biosciences | 742762 |  |
| 2 | CD95 | BV711 | BD Biosciences | 563132 |  |
| 2 | CXCR3 | PE-Cy5 | BD Biosciences | 551128 |  |
| 2 | CXCR5 | BV421 | BD Biosciences | 562747 |  |
| 2 | TACI | PE | BD Biosciences | 558414 |  |
| 2 | BAFF-R | PE-Cy7 | BD Biosciences | 316920 |  |
| 2 | Tbet | BV605 | BioLegend | 644817 | Intra cellular  FoxP3 transcription fix |
| 3 | IgG1 | Biotin & S-BV711 | Miltenyi Biotec | 130-119-858 | Extra cellular  PBS + 4% FCS |
| 3 | IgG3 | FITC | SouthernBiotech | 9210-02 |  |
| 3 | CD32b | AF647 | BioInvent International AB | n/a |  |
| 3 | IFNGR1 | BV650 | BD Biosciences | 743029 |  |
| 3 | FCRL5 | BV605 | BD Biosciences | 749605 |  |
| 3 | IFNa/bR2 | PE-Vio770 | Miltenyi Biotec | 130-099-567 |  |
| 3 | CD69 | PE-Cy5 | BD Biosciences | 555532 |  |
| 3 | TLR7 | PE | R&D Systems | IC5875P | Intra cellular  Cytokine fix |

## Supplementary Figures


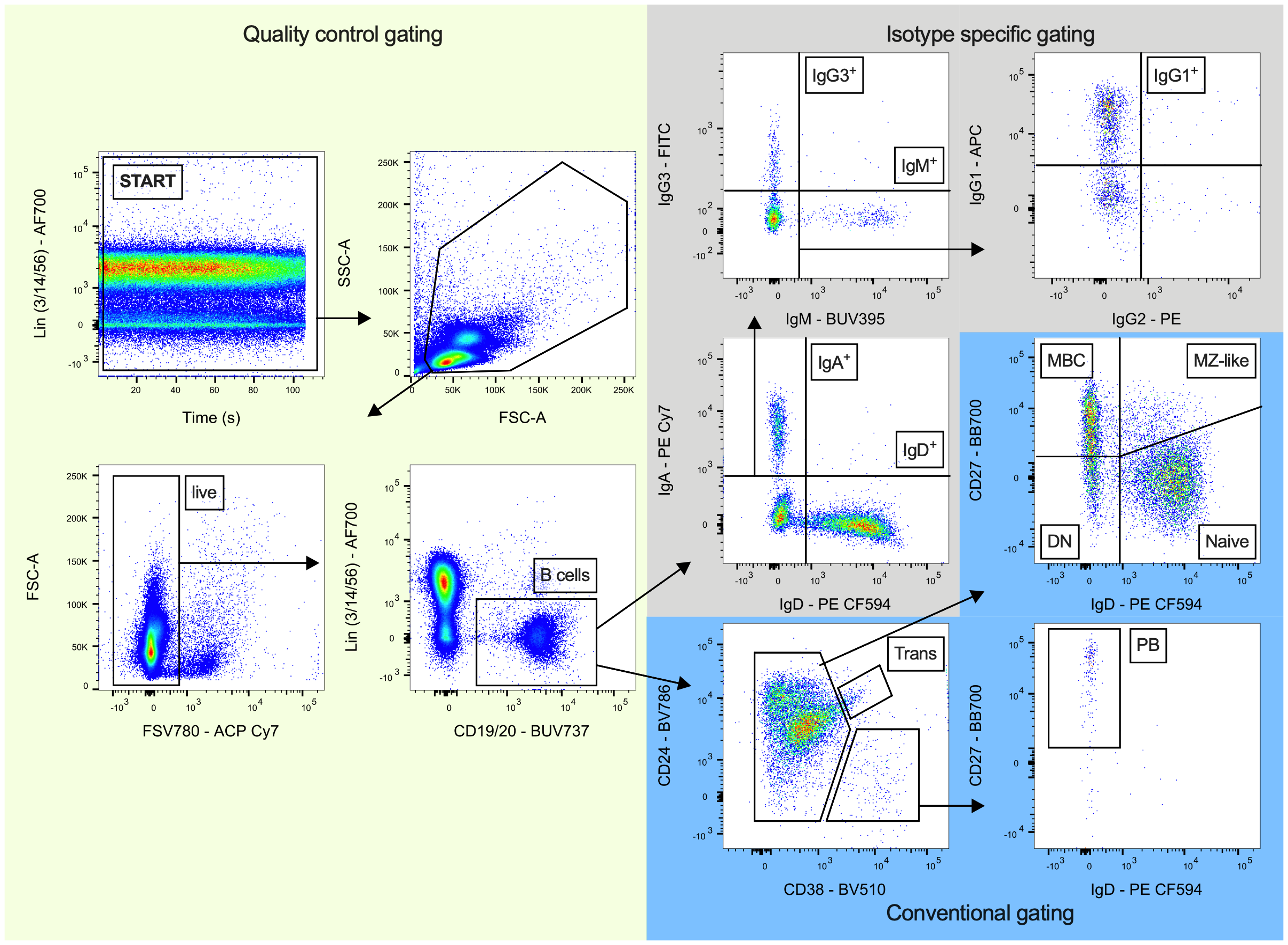


**Supplementary Figure 1.** Gating strategy, including quality controls on acquisition, cell viability. Gating strategy also indicate the isotype specific as well as conventional gating strategy. Data is shown from one representative individual.


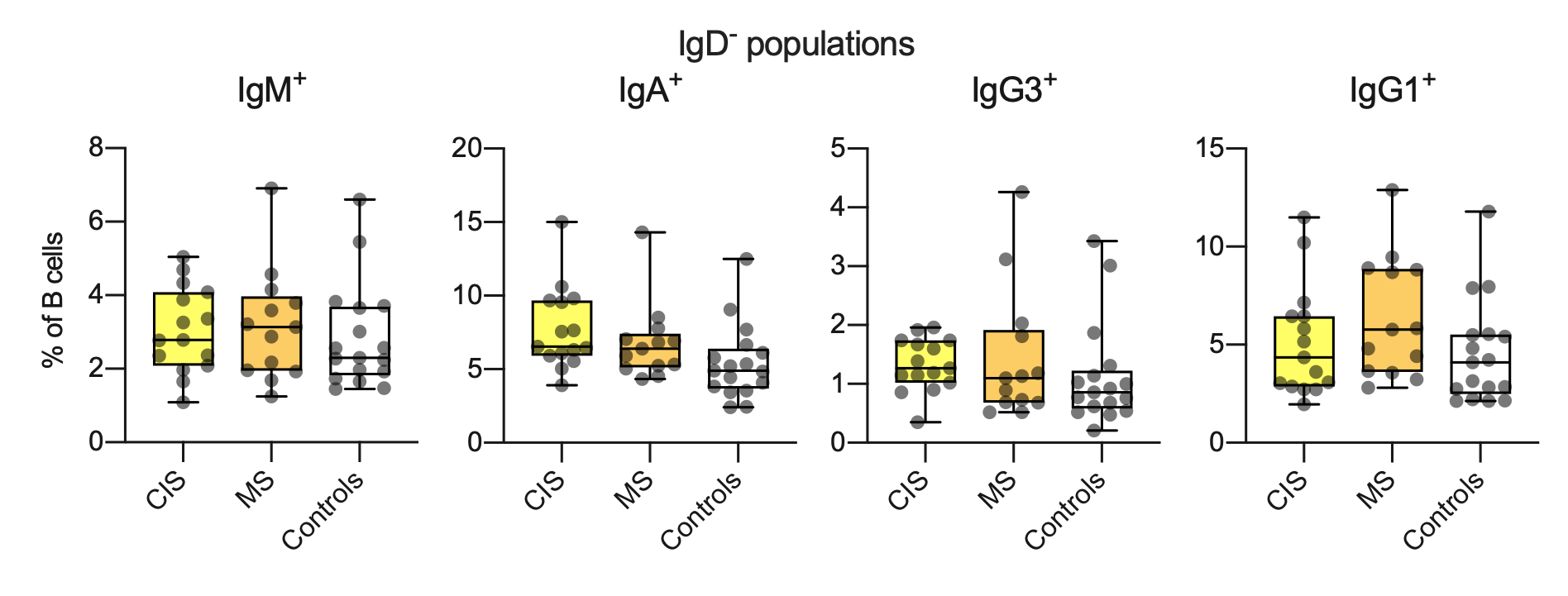


**Supplementary Figure 2.** Abundance of IgM^+^, IgA^+^, IgG3^+^ and IgG1^+^ MBC within the B cell population in CIS and MS patients. Significance of difference between CIS and MS patients (n _CIS_ = 15, n _MS_ = 13, n _Controls_ = 17) was calculated using Mann-Whitney non-parametric test, no significant differences were observed.

**
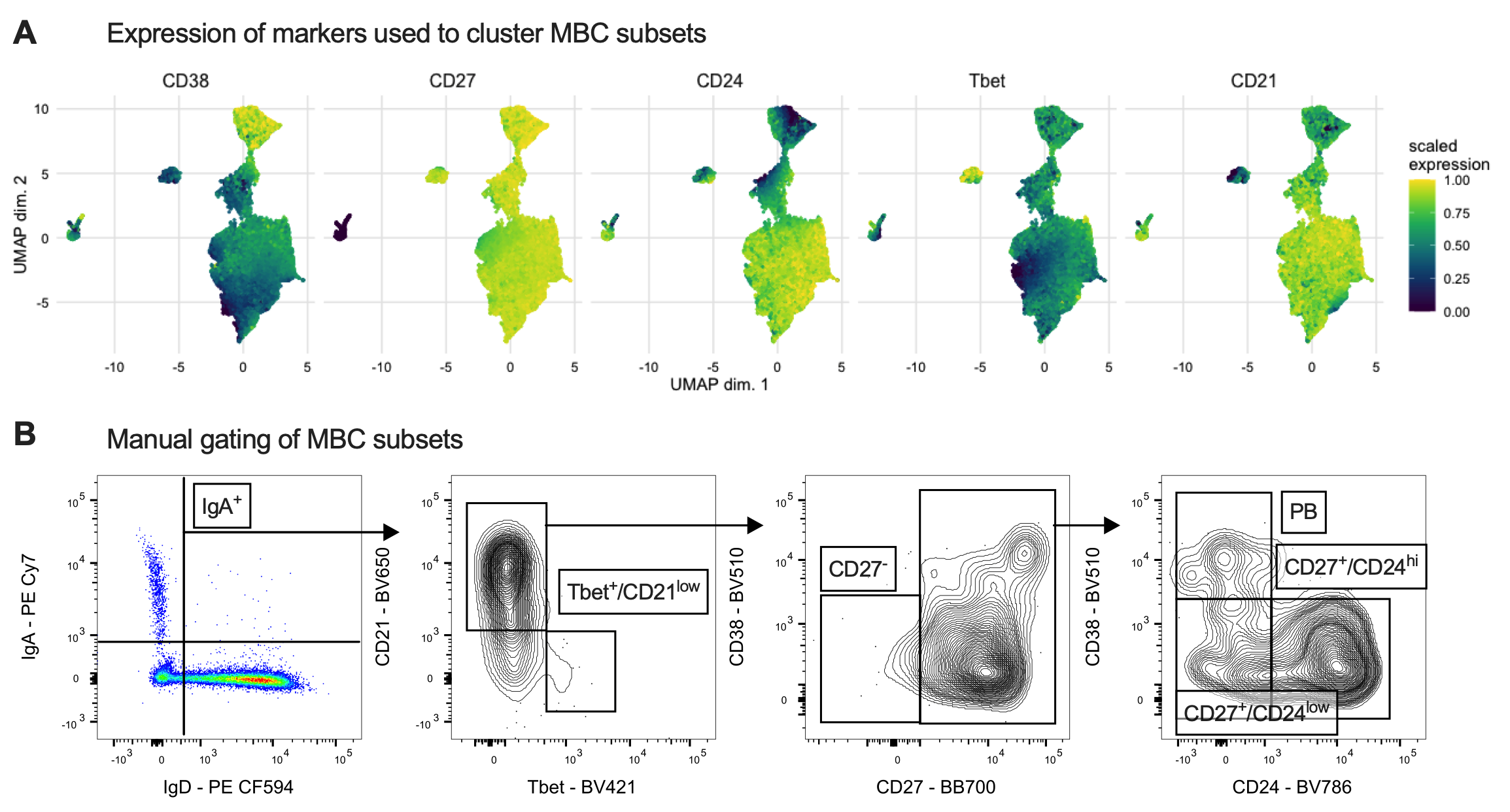
**

**Supplementary Figure 3.** **A** Expression of relevant markers across IgA^+^ MBC used to identify 5 populations across the FlowSOM clusters. **B** Manual gating strategy to identify the same 5 MBC subsets as identified using FlowSOM clustering. In A, mean expression across a subset of individuals (n = 45) is shown, in B, data are from one representative individual.


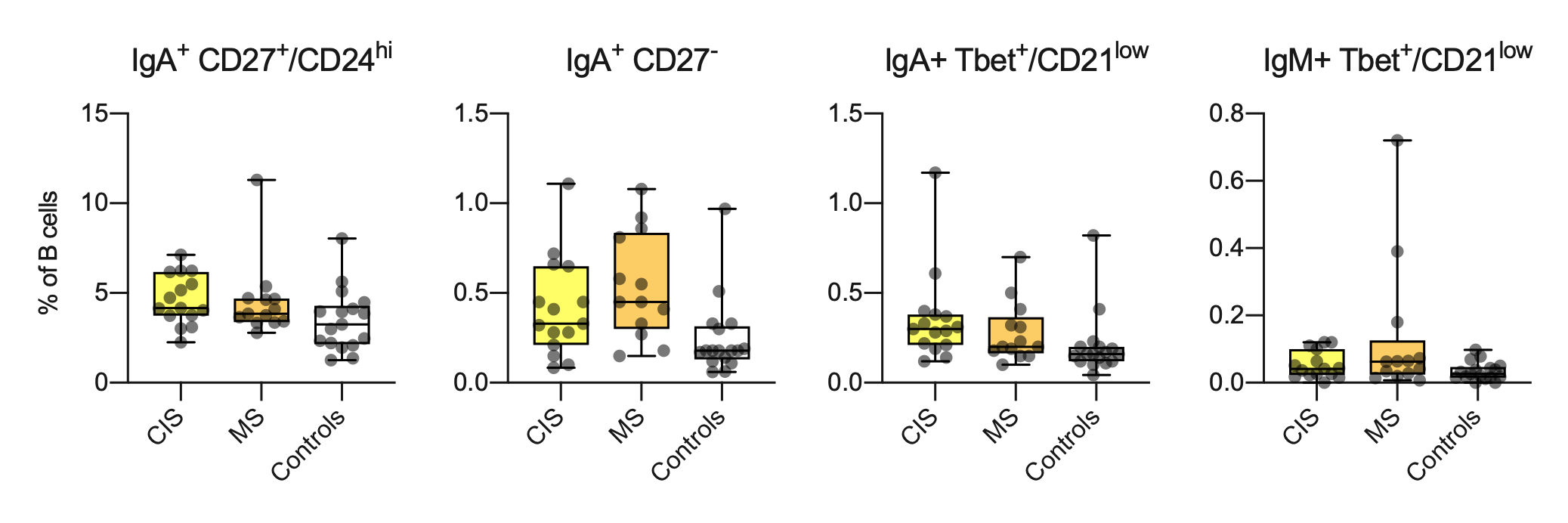


**Supplementary Figure 4.** Abundance of specific MBC subsets that were significantly different in CIS/MS compared to controls, split on CIS and MS patients. Significance of difference between CIS and MS patients (n _CIS_ = 15, n _MS_ = 13, n _Controls_ = 17) was calculated using Mann-Whitney non-parametric test, no significant differences were observed.


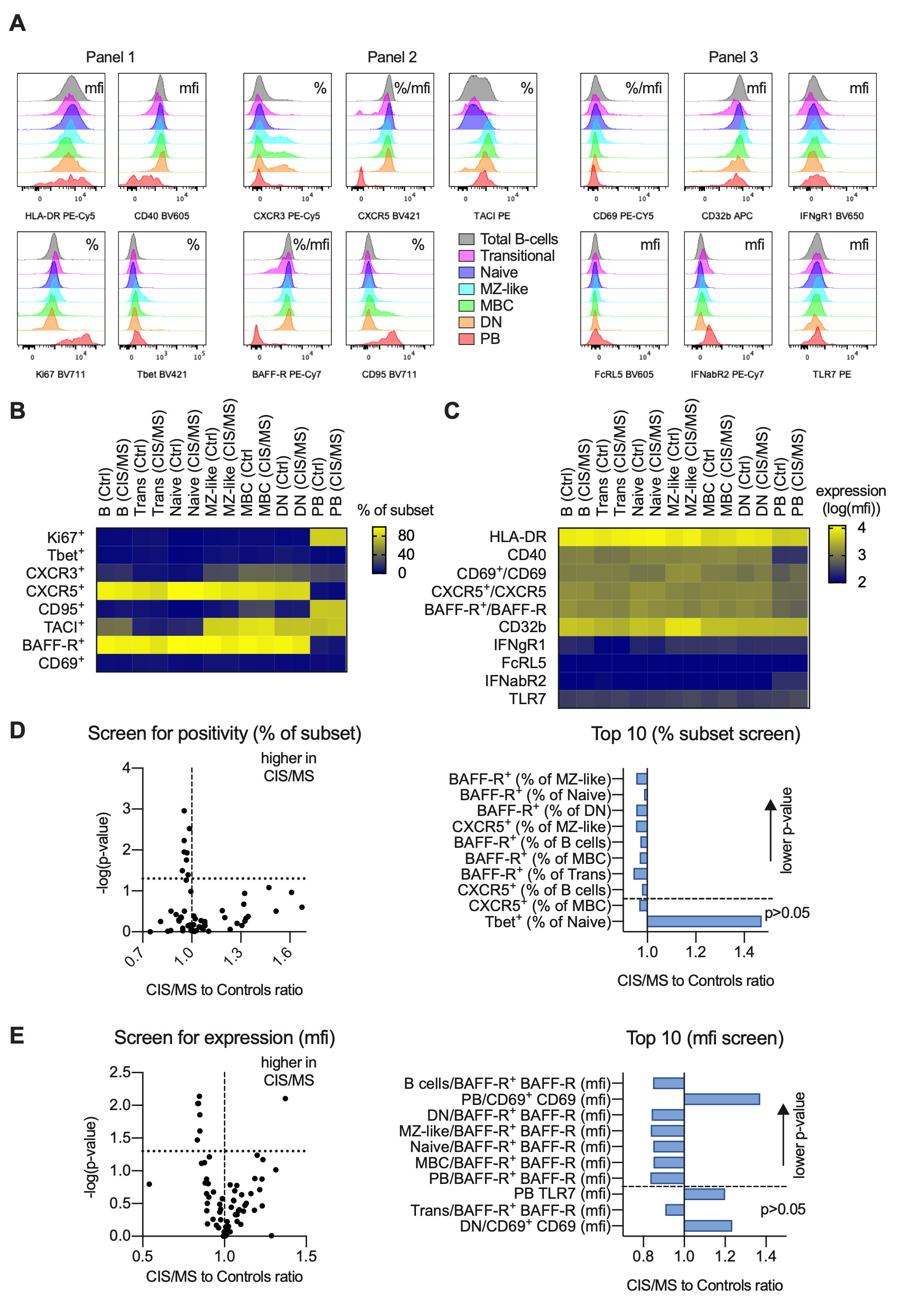


**Supplementary Figure 5.** **A** Expression profile of markers across all B cell subsets from the three B cell panels used to characterise B cell phenotype. Histograms are also labelled to identify if a marker is analysed using expression (mfi) or proportion (%). **B-C** Proportion of positive B cells subsets (**B**) or levels of expression (**C**) across all B cell subsets in CIS/MS patients and controls. **D-E** Ratio of proportion of subsets (**D**) or level of expression (**E**) displayed against significance of finding for CIS/MS patients compared to controls. Ratio of the top 10 most significant findings were also displayed. In A, data is displayed from one representative individual. In B, data is displayed as mean proportion or expression in CIS/MS patients or controls (n _CIS/MS_ = 28, n _Controls_ = 17).


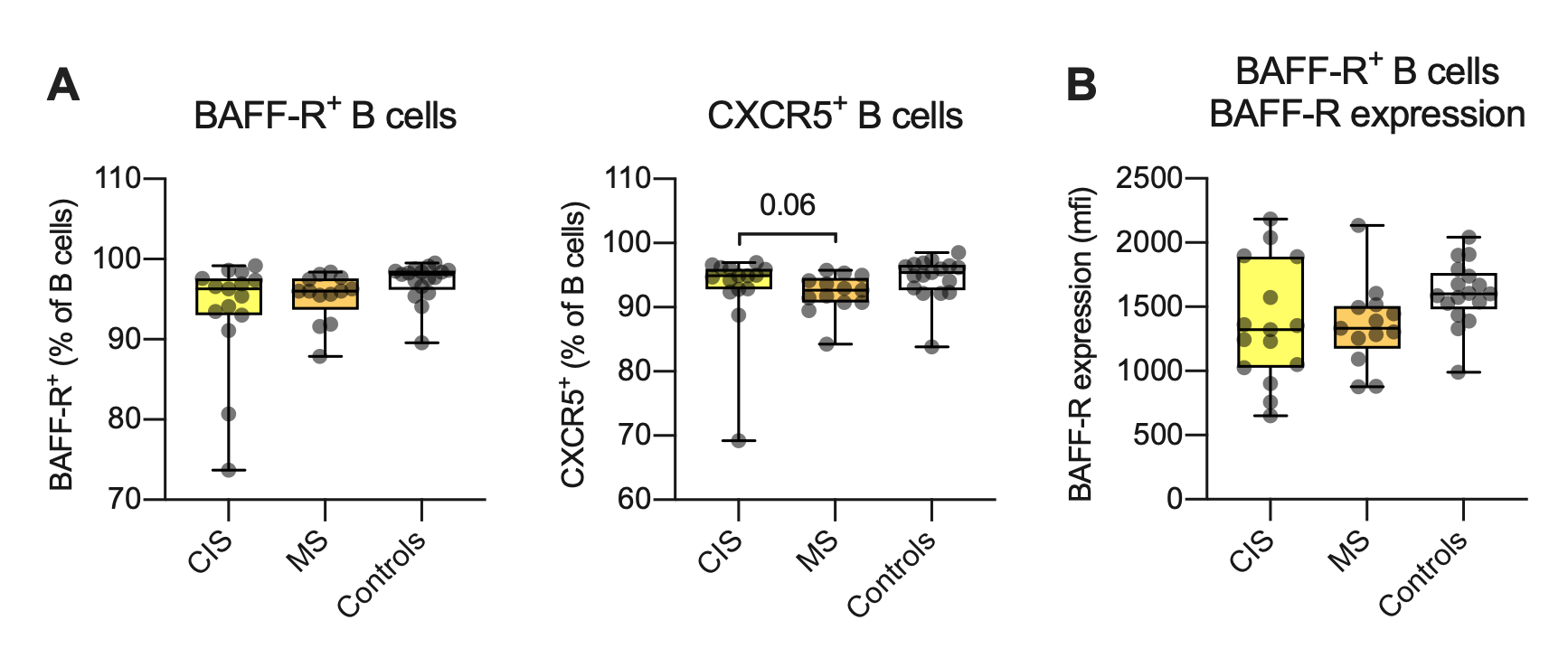


**Supplementary Figure 6.** **A-B** Abundance (**A**) or expression level (**B**) of BAFF-R and/or CXCR5 on total or BAFF-R^+^ B cells in CIS and MS patients. Significance of difference between CIS and MS patients (n _CIS_ = 15, n _MS_ = 13, n _Controls_ = 17) was calculated using Mann-Whitney non-parametric test, no significant differences were observed.


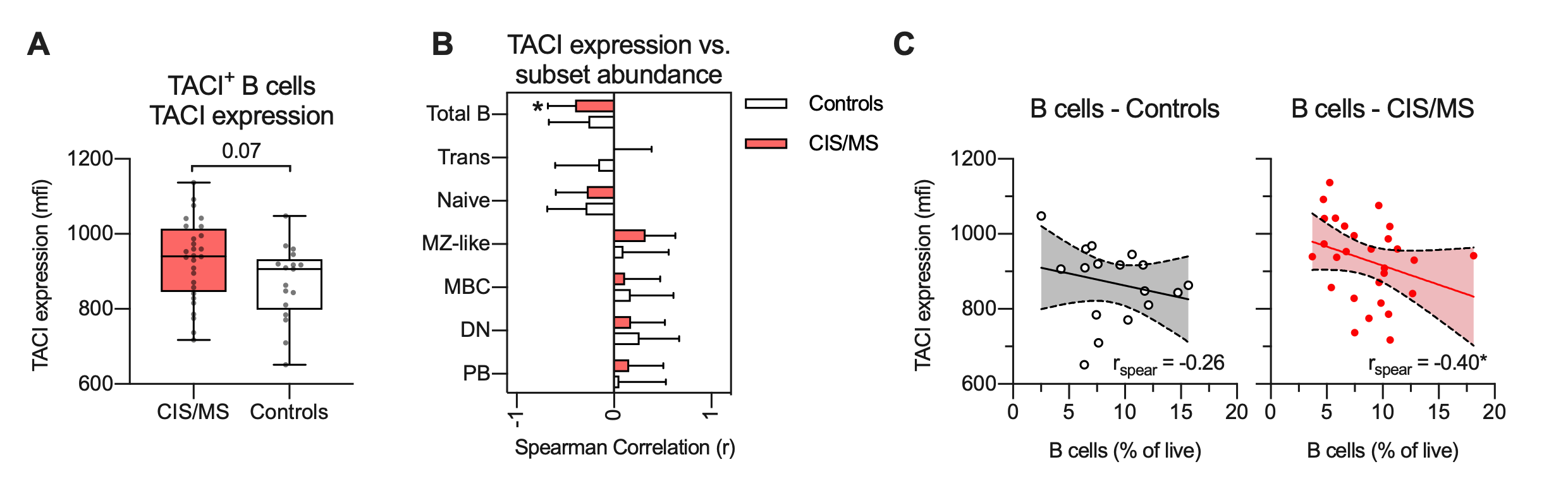


**Supplementary Figure 7.** **A** Expression of TACI on TACI^+^ B cells in CIS/MS and controls. **B-C** Non-parametric correlation between TACI expression on B cells and abundance of B cell subsets in CIS/MS patients and controls (**B**) as well as display of raw data for correlations with abundance of total B cells in CIS/MS and controls (**C**). Data in (A) are displayed for each participant, together with box/whiskers display for median and range. Data in (B) are displayed as mean correlation ± 95% CI or for each participant (n _CIS/MS_ = 28, n _Controls_ = 17) in (C). Correlations are visualised using a linear fit and 95% CI. Significance of difference between CIS/MS versus controls was calculated using Mann-Whitney non-parametric test, significance of correlation was calculated using Spearman non-parametric test and displayed as; p<0.05, *.


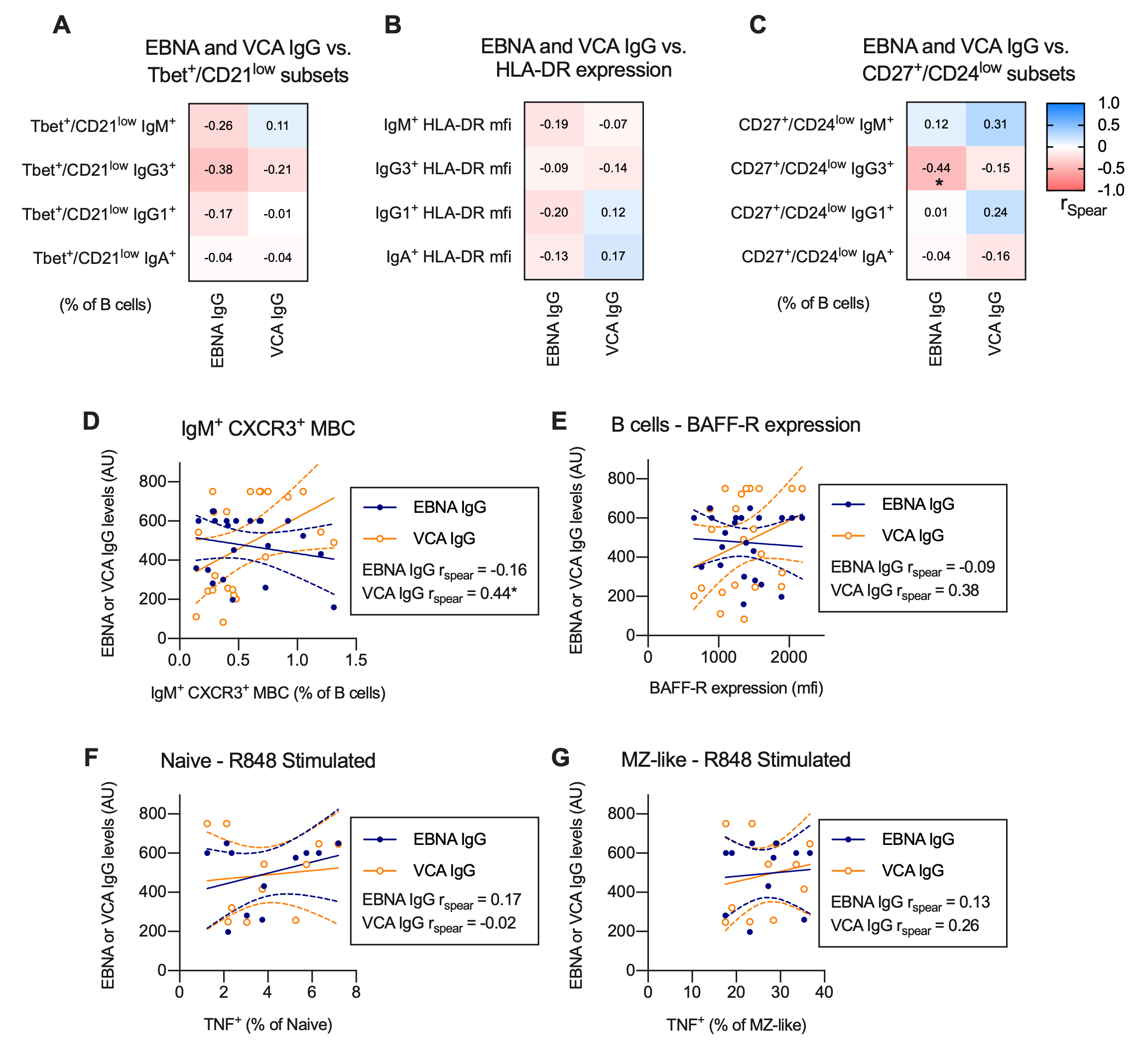


**Supplementary Figure 8.** **A-E** Correlation of Tbet^+^/CD21^low^ MBC (**A**), level of HLA-DR expression (**B**), CD27^+^/CD24^low^ MBC (**C**), IgM^+^ CXCR3^+^ MBC (**D**) and BAFF-R expression on BAFF-R^+^ B cells (**E**) with EBNA and VCA specific IgG titres. **F-G** Correlation of TNF^+^ Naive (**F**) and MZ-like (**G**) cells following R848-stimulation with EBNA and VCA specific IgG titres. In A-C, Spearman’s correlation coefficient is displayed for each comparison. In D-G, data are displayed for each participant and correlations are visualised using a linear fit and 95% CI (n_A-E_ = 22, n_F-G_ = 11). Significance of correlation was calculated using Spearman non-parametric test and displayed as; p<0.05, *.
